# **Global transcriptomic analysis of** ***Francisella tularensis*** **SchuS4 differentially expressed genes in response to doxycycline or ciprofloxacin exposure**

**DOI:** 10.1186/s12863-023-01125-6

**Published:** 2023-04-19

**Authors:** Galia Zaide, Inbar Cohen-Gihon, Ohad Shifman, Ofir Israeli, Moshe Aftalion, Sharon Maoz, Theodor Chitlaru, Raphael Ber, Anat Zvi, Ida Steinberger-Levy

**Affiliations:** Israel Institute for Biological Research, Ness Ziona, Israel

**Keywords:** *Francisella tularensis*, Antimicrobial susceptibility test, Doxycycline, Ciprofloxacin, RNA sequencing, Transcriptomics.

## Abstract

**Objective:**

As part of a research aiming at presenting an alternative approach for rapid determination of antimicrobial susceptibility by quantification of changes in expression levels of specific marker genes and gene sets, cultures of the virulent bacterial strain *Francisella tularensis* SchuS4 were grown in the presence of inhibitory/sub-inhibitory concentrations of either ciprofloxacin or doxycycline and their transcriptomic profiles were elucidated using differential expression analysis followed by functional annotation.

**Data description:**

RNA sequencing was performed to identify differentially expressed genes (DEGs) in response to exposure of *F. tularensis* SchuS4 to either ciprofloxacin or doxycycline, the antibiotics of choice for Tularemia therapy. Accordingly, RNA samples were collected 2 h post antibiotic exposure and subjected to RNA sequence analysis. Transcriptomic quantification of RNA representing duplicated samples generated highly similar gene expression data. Exposure to sub-inhibitory concentration [0.5 x MIC (minimal inhibitory concentration)] of doxycycline or ciprofloxacin modulated the expression of 237 or 8 genes, respectively, while exposure to an inhibitory concentration (1 x MIC) resulted in the modulation of 583 or 234 genes, respectively. Amongst the genes modulated upon doxycycline exposure upregulation of 31 genes encoding for translation-functions could be distinguished, as well as downregulation of 14 genes encoding for functions involved in DNA transcription and repair. Ciprofloxacin exposure impacted differently the RNA sequence profile of the pathogen, resulting in upregulation of 27 genes encoding mainly DNA replication and repair functions, transmembrane transporters and molecular chaperons. In addition, 15 downregulated genes were involved in translation processes.

## Objective

Antibiotic susceptibility tests (AST) are essential for the design of efficient anti-pathogen therapeutic intervention. Yet, as of today, these tests are often time-consuming and may delay appropriate antibiotic treatment. The development of more rapid, accurate ASTs is therefore highly beneficial for prompt medical treatment, and consequently, represents an issue of high public health priority. *F. tularensis* subspecies *tularensis*, the etiological agent of Tularemia, is a highly infectious human pathogen, designated as a Tier-1 select agent [1–3]. Thus, to prevent morbidity and mortality in cases of *F. tularensis* infections, rapid establishment of antibiotic susceptibility exhibited by the infective strain to the recommended antibiotics is needed.

Molecular tests based on monitoring changes in expression levels of marker genes upon antibiotic exposure enable determination of minimal inhibitory concentrations (MICs) within short time frames [4–6]. In the current study, transcriptomic profiling by RNA-Seq analysis was carried out in order to identify *F. tularensis* SchuS4 genes modulated upon exposure to either sub-inhibitory or inhibitory concentrations of ciprofloxacin or doxycycline. The data presented here provides a detailed picture of the *F. tularensis* global response to doxycycline and ciprofloxacin and establishes the expression signatures of the interaction between *F. tularensis* and these antimicrobial agents. Some of the differentially expressed genes may serve as markers for the design of future *F. tularensis* susceptibility tests.

## Data description

Here we report a comprehensive RNA-Seq transcriptomic study generating a database of differentially expressed genes (DEGs) upon exposure to sub-inhibitory as well as inhibitory concentrations of either doxycycline or ciprofloxacin. Samples were prepared by growing *F*. *tularensis* SchuS4 strain, in duplicates, in the presence or absence of 0.5 x MIC or 1 x MIC of either doxycycline or ciprofloxacin, collected 2 h post exposure and subjected to total RNA purification. Library construction was carried out using Illumina TruSeq chemistry. The RNA libraries were sequenced at the JP Sulzberger Columbia Genome Center. Mapping was performed using Bowtie 2 [7] and the expression level of each gene was determined using HTSeq [8]. Analysis of DEGs under various conditions was conducted using the R package DESeq2 [9]. RNA-Seq analysis of two independent duplicated experimental groups resulted in a consistent and highly similar gene expression data. Analysis of the DEGs was based on comparing the expression level of genes originating from antibiotic-treated samples to genes emerging from untreated control samples [10]. The transcriptomic data have been deposited to the NCBI Gene Expression Omnibus (GEO) database [11–24].

Exposure to sub-inhibitory concentration (0.5 x MIC) of either doxycycline or ciprofloxacin modulated the expression level of 237 or 8 genes, respectively. Exposure to 1 x MIC inhibitory concentration of doxycycline resulted in the modulation of 583 genes, while 234 genes were modulated upon exposure to 1 x MIC concentration of ciprofloxacin [25]. Fifty-eight of the 1 x MIC responsive genes were modulated upon exposure to both antibiotics.

Modulation of expression upon exposure to 0.5- or 1 x MIC concentrations of either doxycycline or ciprofloxacin was relatively moderate: only a small fraction of genes (less than 5%) were modulated over 3-fold upon exposure to either antibiotic treatment.

Exposure to 0.5 x MIC of doxycycline induced the expression of 124 genes, out of which 35 genes (28%) encode ribosomal subunits (26). In addition, 12 out of the 113 downregulated genes (9%) encode mainly DNA repair functions (27). Exposure to sub-inhibitory concentration of ciprofloxacin (0.5 x MIC) resulted in a small number of modulated genes (25). Yet, it is worth noting that the gene encoding the FTT_1400c protein was upregulated 5-fold upon exposure to 0.5 MIC ciprofloxacin. Of the 136 genes upregulated upon exposure to 1 x MIC of ciprofloxacin, 27 genes (20%) encode various ATP binding processes, among which are transmembrane transporters, DNA repair enzymes, nucleotide synthesis proteins and molecular chaperons (28). Translation emerged as the main downregulated function as 15 out of the 98 downregulated genes (15%) encode ribosomal proteins (29).

To conclude, this study generated a comprehensive database of differentially expressed genes representing the transcriptomic signature that stems from exposure of *F. tularensis* SchuS4 to either doxycycline or ciprofloxacin. Together with further interrogation of the functions and the metabolic pathways that are overexpressed upon exposure to these antibiotics, the results might pave the way towards the development of a molecular AST based on quantifying alteration in expression levels of antibiotic-responsive mRNAs markers and metabolic pathways. Please refer to Table 1 for links to Data files 1–7 and Data sets 1–12.

### Limitations

- Exposure to 0.5 x MIC of ciprofloxacin resulted in a small number of modulated genes. Therefore, ciprofloxacin susceptibility marker genes could mainly be identified among the genes modulated upon exposure to 1 x MIC concentration. However, antibiotic treatment at MIC concentrations affects the growth rate and thus nonspecifically influences the transcription level of many genes. Thus, caution should be exerted while considering the modulation of such genes as a specific regulatory response to the antibiotic treatment.
- Validation of potential marker genes should be carried out by comparing the changes in expression level of these markers to their modulation level in mutant strains exhibiting reduced susceptibility to the corresponding antibiotic.
- For the development of rapid molecular-AST we examined the alterations in gene expression occurring upon SchuS4 exposure to antibiotic at one time point (2 h) and according to the CLSI recommended standard growth conditions. As transcriptomic processes are dynamic and might differ in response to various strains, growth media etc., the universality of the presented transcriptomic changes has to be tested in additional *F. tularensis* strains.

**Table 1:** Overview of data files/data sets

Label	Name of data file/data set	File type (file extension)	Data repository and identifier (DOI or accession number)
Data file 1	Materials and methods	Portable Document Format file (.pdf)	10.6084/m9.figshare.20971000 [10]
Data file 2	Number of genes modulated upon exposure to doxycycline or ciprofloxacin	Portable Document Format file (.pdf)	10.6084/m9.figshare.20934631 [25]
Data file 3	Genes upregulated upon exposure to doxycycline	Spreadsheet (.xls)	10.6084/m9.figshare.20938612.v1 [26]
Data file 4	Genes downregulated upon exposure to doxycycline	Spreadsheet (.xls)	10.6084/m9.figshare.20938786.v1 [27]
Data file 5	Genes upregulated upon exposure to ciprofloxacin	Spreadsheet (.xls)	10.6084/m9.figshare.20938972.v1 [28]
Data file 6	Genes downregulated upon exposure to ciprofloxacin	Spreadsheet (.xls)	10.6084/m9.figshare.20939176.v1 [29]
Data file 7	Count table samples	Text file (.txt)	https://identifiers.org/geo:GSE210974 [12]
Data set 1	* F. tularensis* without treatment with doxycycline 1st duplicate	SRA file (.sra)	https://identifiers.org/ncbi/insdc.sra:SRX17025608 [11]
Data set 2	* F. tularensis* without treatment with doxycycline 2nd duplicate	SRA file (.sra)	https://identifiers.org/ncbi/insdc.sra:SRX17025609 [13]
Data set 3	* F. tularensis* without treatment with ciprofloxacin 1st duplicate	SRA file (.sra)	https://identifiers.org/ncbi/insdc.sra:SRX17025610 [14]
Data set 4	* F. tularensis* without treatment with Ciprofloxacin 2nd duplicate	SRA file (.sra)	https://identifiers.org/ncbi/insdc.sra:SRX17025611 [15]
Data set 5	* F. tularensis* 2 h post exposure to 0.5xMIC Doxycycline 1st duplicate	SRA file (.sra)	https://identifiers.org/ncbi/insdc.sra:SRX17025612 [16]
Data set 6	* F. tularensis* 2 h post exposure to 0.5xMIC Doxycycline 2nd duplicate	SRA file (.sra)	https://identifiers.org/ncbi/insdc.sra:SRX17025613 [17]
Data set 7	* F. tularensis* 2 h post exposure to 1xMIC Doxycycline 1st duplicate	SRA file (.sra)	https://identifiers.org/ncbi/insdc.sra:SRX17025614 [18]
Data set 8	* F. tularensis* 2 h post exposure to 1xMIC Doxycycline 2nd duplicate	SRA file (.sra)	https://identifiers.org/ncbi/insdc.sra:SRX17025615 [19]
Data set 9	* F. tularensis* 2 h post exposure to 0.5xMIC Ciprofloxacin 1st duplicate	SRA file (.sra)	https://identifiers.org/ncbi/insdc.sra:SRX17025616 [20]
Data set 10	* F. tularensis* 2 h post exposure to 0.5xMIC Ciprofloxacin 2nd duplicate	SRA file (.sra)	https://identifiers.org/ncbi/insdc.sra:SRX17025617 [21]
Data set 11	* F. tularensis* 2 h post exposure to 1xMIC Ciprofloxacin 1st duplicate	SRA file (.sra)	https://identifiers.org/ncbi/insdc.sra:SRX17025618 [22]
Data set 12	* F. tularensis* 2 h post exposure to 1xMIC Ciprofloxacin 2nd duplicate	SRA file (.sra)	https://identifiers.org/ncbi/insdc.sra:SRX17025619 [23]

## Data Availability

Data files 1–6 described in this data note can be freely and openly accessible on Figshare (https://figshare.com/) [10, 25–29]. Data sets 1–12 [11, 13–23] and data file 7[12] are available on the NCBI database. The raw reads as well as the count table were submitted to the NCBI Gene Expression Omnibus under the accession number GSE210974 (https://identifiers.org/geo:GSE210974).

## Abbreviations

MIC: Minimal Inhibitory Concentration
DEGs: Differentially Expressed Genes
RNA -Seq analysis: RNA Sequence Analysis
GEO: Gene Expression Omnibus
